# Editorial

**DOI:** 10.1155/S1357714X00000013

**Published:** 2000-03

**Authors:** M. H. Robinson


*Sarcoma* (2000) **4**, 5

### Editorial

*Sarcoma* has now been published for three years. We hope that we have gone some way towards providing a forum for all researchers and clinicians in the field of sarcomas. I would like to encourage further discussion in this area and, to this end, we are publishing a summary of the proceedings of the meeting of the Canadian Sarcoma Group. Dr Vivien Bramwell has very kindly provided this and it gives us a valuable insight into the potential areas for research and development, both scientifically and clinically. The Canadian Sarcoma Group are an excellent example of how a National Consensus can be arrived at and result in extremely valuable scientific and clinical research. This is demonstrated most clearly by the results of the last radiotherapy trial. This demonstrated that the use of pre-operative radiotherapy is associated with increased wound complication when compared to post-operative radiotherapy. It does not however mean that pre-operative radiotherapy is inappropriate in all cases. I await the final results of this trial with interest.

We also include a section listing papers published in other journals this year which may be of interest to our readers. We propose to continue to provide this service and I hope to provide comments alongside some of these to help our readers pick out the articles of interest to themselves.

We continue to strive to improve this journal and I would be grateful for any comments, suggestions and manuscripts which might help further our aims in this direction. On this note we have reached agreement with EMSOS to publish the abstracts of their last meeting and future meetings. In our next issue we hope to publish the proceedings of the UKCCCR Sarcoma Group and, in the future, the proceedings of the EORTC 2001 Anniversary Conference.

*Dr M. H. Robinson*
*Editor*

1357-714X print/1369-1643 online/00/010005-01 © 2000 Taylor & Francis Ltd

